# Binding of Staphylococcal Enterotoxin B (SEB) to B7 Receptors Triggers TCR- and CD28-Mediated Inflammatory Signals in the Absence of MHC Class II Molecules

**DOI:** 10.3389/fimmu.2021.723689

**Published:** 2021-08-13

**Authors:** Martina Kunkl, Carola Amormino, Silvana Caristi, Valentina Tedeschi, Maria Teresa Fiorillo, Revital Levy, Andrey Popugailo, Raymond Kaempfer, Loretta Tuosto

**Affiliations:** ^1^Department of Biology and Biotechnology Charles Darwin, Sapienza University, Rome, Italy; ^2^Laboratory Affiliated to Istituto Pasteur Italia-Fondazione Cenci Bolognetti, Sapienza University, Rome, Italy; ^3^Department of Biochemistry and Molecular Biology, The Institute for Medical Research Israel-Canada, The Hebrew University-Hadassah Medical School, Jerusalem, Israel

**Keywords:** staphylococcal enterotoxins, inflammatory response, T cells, CD28, TCR - T cell receptor

## Abstract

The inflammatory activity of staphylococcal enterotoxin B (SEB) relies on its capacity to trigger polyclonal T-cell activation by binding both T-cell receptor (TCR) and costimulatory receptor CD28 on T cells and MHC class II and B7 molecules on antigen presenting cells (APC). Previous studies highlighted that SEB may bind TCR and CD28 molecules independently of MHC class II, yet the relative contribution of these interactions to the pro-inflammatory function of SEB remained unclear. Here, we show that binding to MHC class II is dispensable for the inflammatory activity of SEB, whereas binding to TCR, CD28 and B7 molecules is pivotal, in both human primary T cells and Jurkat T cell lines. In particular, our finding is that binding of SEB to B7 molecules suffices to trigger both TCR- and CD28-mediated inflammatory signalling. We also provide evidence that, by strengthening the interaction between CD28 and B7, SEB favours the recruitment of the TCR into the immunological synapse, thus inducing lethal inflammatory signalling.

## Introduction

*Staphylococcus aureus* is a gram-positive bacterium that colonizes human epithelia and soft tissues, causing food poisoning and systemic intoxication that can lead to a severe life-threatening condition known as toxic shock syndrome (TSS) (1). Among the virulence factors, *Staphylococcus aureus* secretes toxins that act as superantigens (SAgs) and stimulate a large number of T cells to produce inflammatory cytokines (2). Such SAgs include the toxic shock syndrome toxin-1 (TSST-1) and the staphylococcal enterotoxins (SE) A-Z (3). SEB was one of the first SAgs to be identified (4) and, together with SEA and SED, is a major cause of food poisoning and TSS (5). Like TSST-1 and other SEs and the related streptococcal pyrogenic exotoxins, even at extremely low concentrations SEB activates a large proportion of T lymphocytes (up to 30%), thereby inducing a vastly excessive of inflammatory cytokines including IL-2, TNF, IFN-γ and IL-17A that can lead to respiratory failure, vascular damage, multi-organ system breakdown and death (5, 6).

The superantigen activity of SEB relies on its ability to interact with the outer leaflet of MHC class II molecules, in particular HLA-DR, on antigen presenting cells (APCs) and specific elements within the variable domain of the TCR β-chain (Vβ), thus inducing the polyclonal activation of T cells and cytokine storm (7–10). The SEB/TCR/MHC class II ternary complex formed outside the TCR and MHC class II peptide-binding domains is also able to circumvent the restriction between the TCR and the peptide bound to MHC class II (11, 12). Recent findings demonstrated that to elicit inflammatory cytokine production, SEB must also bind directly to the costimulatory receptor CD28 and its natural coligands B7.1/CD80 and B7.2/CD86 (13–16). CD28 is an important costimulatory receptor constitutively expressed as a homodimer on T cell surface that binds B7.1/CD80 and B7.2/CD86 on APCs and cooperates with the TCR to induce optimal T cell activation (17, 18). Moreover, human CD28 is also able to elicit TCR-independent signals when engaged by B7 molecules, which favour T cell:APC conjugate formation, cytoskeleton reorganization and cytokine/chemokine production (19–22). CD28-dependent expression and production of inflammatory cytokines and chemokines by SEB depends on a 12 amino-acid β-strand(8)/hinge/α-helix(4) domain that is highly conserved in bacterial SAgs (23), which specifically engages the homodimer interfaces of CD28 and B7 coligands, thus enhancing their interaction (13, 16, 24). Short peptide mimetics of the CD28 homodimer interface are able to dampen inflammatory cytokine production induced by SEB and by diverse SAgs both *in vitro* and *in vivo* (13, 16, 25–27), thus demonstrating the crucial role of the CD28/B7 costimulatory axis.

Notably, the region of SEB involved in binding of CD28 and B7 molecules is distal from TCR and MHC class II binding sites (23). Indeed, SEB is able to interact directly with CD28 and B7 molecules also in the absence of MHC class II or other coligands (13–16, 24). However, the functional relevance of this interaction without MHC class II still remains unknown.

In this study, we verified that the stimulation of the TCR and CD28/B7 costimulatory axis by SEB suffices to elicit an inflammatory cytokine response even in the absence of MHC class II. Our data in both human primary CD4^+^ and CD8^+^ T cells and Jurkat T cell lines demonstrate that binding of SEB to MHC class II molecules is dispensable for SEB-mediated inflammatory cytokine production, whereas TCR- and CD28-mediated signalling are mandatory. In accordance with the known ability of SEB to enhance CD28/B7 engagement (16), we found that SEB significantly increases the interaction between T cells and B7-expressing APCs and promotes TCR recruitment at the T:APC interface. Our findings suggest that, by enhancing CD28/B7 engagement and by binding simultaneously to CD28 and the TCR, SEB may favour the recruitment of the TCR into the immunological synapse, thus inducing the activation of inflammatory signals.

## Materials and Methods

### Cells, Antibodies, and Reagents

Human primary CD4^+^ and CD8^+^ T cells were enriched from peripheral blood mononuclear cells (PBMC) by negative selection using a EasySepTM isolation kit (#17952) (STEMCELL Technology, CAN) and positive selection using MACS microbeads sorting kit (#130-045-201, Miltenyi Biotec, Milan, Italy), respectively, and cultured in RPMI 1640 supplemented with 5% human serum (Euroclone, UK), L-glutamine, penicillin and streptomycin. The purity of the sorted population was 95-99%, as evidenced by staining with anti-CD3 plus anti-CD4 or anti-CD8 Abs. PBMCs were derived from buffy coats of anonymous healthy blood (HD) donors provided by the Policlinico Umberto I (Sapienza University of Rome, Italy). Written informed consent was obtained from blood donors and both the informed consent form and procedure were approved by the Ethics Committee of Policlinico Umberto I (ethical code N. 1061bis/2019, 13/09/2019).

CD28-negative Jurkat T cell line CH7C17 (28), CH7C17 cells, stably transfected with human CD28WT (29) and TCR-negative 31.13 Jurkat T cell line (30) were maintained in culture as previously described (21, 31). Murine L cells (Dap3) or Dap3 cells transfected with human B7.1/CD80 (Dap3/B7) (32), or murine L cells co-transfected with HLA-DRB1*0101 and B7.1/CD80 (5-3.1/B7) (33, 34) were used as previously described (22).

The following antibodies were used: anti-human CD80-FITC (#21270803), anti-human CD86-FITC (#21480863), anti-human CD4-PE (#21278044), anti-human CD4-FITC (#21850043), anti-human CD8-APC (#21620086) (Immunotools, Germany); anti-human CD8-FITC (#555634), anti-human CD28-FITC (#556621), anti-human CD86-PE (#555658), anti-human CD3-PE (#555333) (BD Biosciences, Italy); anti-human HLA-DR-APC (#130-095-297), anti-human CD28-PE (#130-109-441) (Miltenyi Biotec, Italy); superagonistic anti-human CD28 (ANC28.1, #177820) (Ancell, USA); rabbit anti-GAPDH (#sc-25778), rabbit anti-HA (Y11, #sc-805) (Santa Cruz Biotechnology, USA); sheep anti-SLP76 (#06-548), mouse anti-pTyr (#05-321, Merck-Millipore, Italy); mouse anti-human CD3 (UCHT1, #555330), goat anti-mouse Alexa-flour 594 (#A11020), phalloidin-633 (#A22284) (ThermoFisher Scientific, Italy). Staphylococcal Enterotoxin A (SEA, #59399) and Staphylococcal Enterotoxin B (SEB, #54881) were purchased by Merck (Italy).

### Plasmids, Cell Transfection and Luciferase Assays

The NF-κB luciferase gene under the control of six thymidine kinase NF-κB sites (35), IL-2 luciferase construct (36), NF-AT luciferase reporter construct containing the luciferase gene under the control of the human IL-2 promoter NF-AT binding site (37) and AP-1-luciferase construct containing the luciferase gene under the control of two human collagenase TRE sites (38) were used.

HA-Nck R311K mutant construct was provided by Wei Li (University of Southern California, Los Angeles, CA, USA). pEF-Bos encoding Flag tagged SLP-76 and dominant-negative mutant of SLP-76 bearing mutations of Y113, Y128 and Y145 to phenylalanine (39) were provided by G. A. Koretzky (Weill Cornell Medicine, New York, USA).

For luciferase assays, 10^7^ cells were electroporated at 260 V, 960 µF in 0.5 ml RPMI 1640 medium supplemented with 10% FBS. For transfections, 2 µg NF-κB luciferase or 10 µg NF-AT luciferase or IL-2 luciferase or AP-1 luciferase constructs together with 5 µg pEGFP and each indicated expression vector were used, keeping constant the total amount of DNA (40 µg) with empty vector. At 24 hours after transfection, cells were stimulated with Dap3 or Dap/B7 or 5-3.1/B7 cells in the presence or absence of SEB or SEA (1 µg ml^-1^) at 37°C for 6 hours. Luciferase activity was measured according to the manufacturer’s instruction (Promega). Luciferase activity determined in triplicates was expressed as arbitrary luciferase units (AU) or fold inductions (F.I.) after normalization to GFP values.

### Cell Stimulation and Immunoblotting

Jurkat T cell lines were stimulated with 1 µg ml^-1^ SEB for different times at 37°C. At the end of incubation, cells were harvested and lysed for 30 min on ice in 1% Nonidet P-40 lysis buffer in the presence of inhibitors of proteases and phosphatases. Proteins were resolved by SDS-PAGE and blotted onto nitrocellulose membranes. Blots were incubated with the indicated primary antibodies, extensively washed and after incubation with horseradish peroxidase (HRP)-labelled goat anti-rabbit (#NA934V), HRP-labelled goat anti-mouse (#NA931V) (Amersham), or (HRP)-labelled donkey anti-sheep Abs (#sc-2473) (Santa Cruz Biotechnology) were developed with the enhanced chemiluminescence detection system (GE Healthcare, Italy).

### Cytokine Production

Secretion of IL-8, IL-2, TNF, IFN-γ, IL-6 and IL-17A was measured in the supernatants of Jurkat T cell lines or T cells cultured for 24 hours in flat-bottom 24-culture wells (2 x 10^6^ cells per well) either unstimulated or stimulated with 1 µg ml^-1^ SEB or SEA in the presence or absence of adherent Dap3 or Dap3/B7 cells (10^6^ cells per well) by using human IL-8 (#DY208-05), IL-2 (#DY202-05), TNF (#DY210-05), IFN-γ (#DY285B-05), IL-6 (#DY206-05) and IL-17A (#DY317-05) ELISA kits (R&D Systems). Data were analysed by a Bio-Plex (Bio-Rad, Hercules, CA, USA). The assays were performed in duplicate. The sensitivity of the assay was 9.4 pg ml^-1^ for IL-6 and IFN-γ, 15.6 pg ml^-1^ for IL-2, TNF and IL-17A, and 31.3 pg ml^-1^ for IL-8.

### Measurement of Intracellular Calcium Concentration

CH7C17, CD28WT, or 31.13 Jurkat T cell lines (2 x 10^6^ ml^-1^) were loaded with 20 μM Fluo-3 AM (Sigma) for 30 min at 37°C in 400 μl RPMI 1640. Loaded cells were then washed and activated with SEB at 37°C and immediately analysed by a flow cytometer (FACScalibur, BD Biosciences, Italy). Changes in cell fluorescence was monitored every 30 sec for 10 min by measuring fluorescence emission at 530 nm. The concentration of intracellular calcium [Ca^2+^]_i_ was calculated according to Grynkiewicz et al. (40).

### Measurement of Conjugate Formation

Conjugate formation was measured as described previously (22). Briefly, CH7C17, CD28WT, or 31.13 cells (1 × 10^6^) were incubated for 20 min at 37°C with Dap3 or Dap3/B7 cells (1 × 10^6^) in the presence or absence of SEB in a final volume of 40 μl RPMI 1640, then diluted in 500 μl RPMI and analysed by FACS. Conjugates were identified on a total of 10^5^ events by gating for SSC and FSC and expressed as the mean percentage ± SEM of triplicate samples.

### Confocal Microscopy

15x10^3^ murine Dap3/B7 cells were adhered on cover glasses (12 mm) overnight at 37°C. CD28WT or CH7 cells were seeded on cover glasses for 20 min at 37°C, fixed by 2% paraformaldehyde and permeabilized by 0.1% saponin in PBS containing 1% BSA. CD3 staining was performed by using anti-human CD3 (UCHT1) followed by goat anti-mouse Alexa-flour 594. Filamentous actin (F-Actin) was stained by using phalloidin-633. Confocal observations were performed using a Leica DMIRE (Leica Microsystems, Heidelberg, Germany) and a Zeiss LSM 780 camera (Zeiss, Berlin, Germany). Images were analysed with the Adobe® Photoshop® program. The relative recruitment index (RRI) was calculated as previously described (41) by the formula: RRI = [mean fluorescence intensity (MFI) at synapse – background]/[MFI at all the cell membrane not in contact with APC – background]. At least thirty cells or conjugates were examined quantitatively for each experiment. Statistical significance was calculated using a Student’s *t* test. Signals from different fluorescent probes were taken in parallel. Several cells were analysed for each labelling condition, and representative results are shown.

### Statistical Analysis

The sample size was chosen based on previous studies to ensure adequate power. Parametrical statistical analysis (mean and standard deviation) was performed to evaluate differences between continuous variables through Prism 8.0 (GraphPad Software, San Diego, CA), using standard unpaired *t* test or two-way ANOVA with Dunnett test for comparisons. For multiple group comparisons, significant differences were calculated using the nonparametric Mann–Whitney *U* test, and linear regression analyses were performed using the Pearson chi-squared test. For all tests, P values < 0.05 were considered significant.

## Results

### MHC Class II Molecules Are Dispensable for SEB-Induced Inflammatory Signals

**Figure 1:**
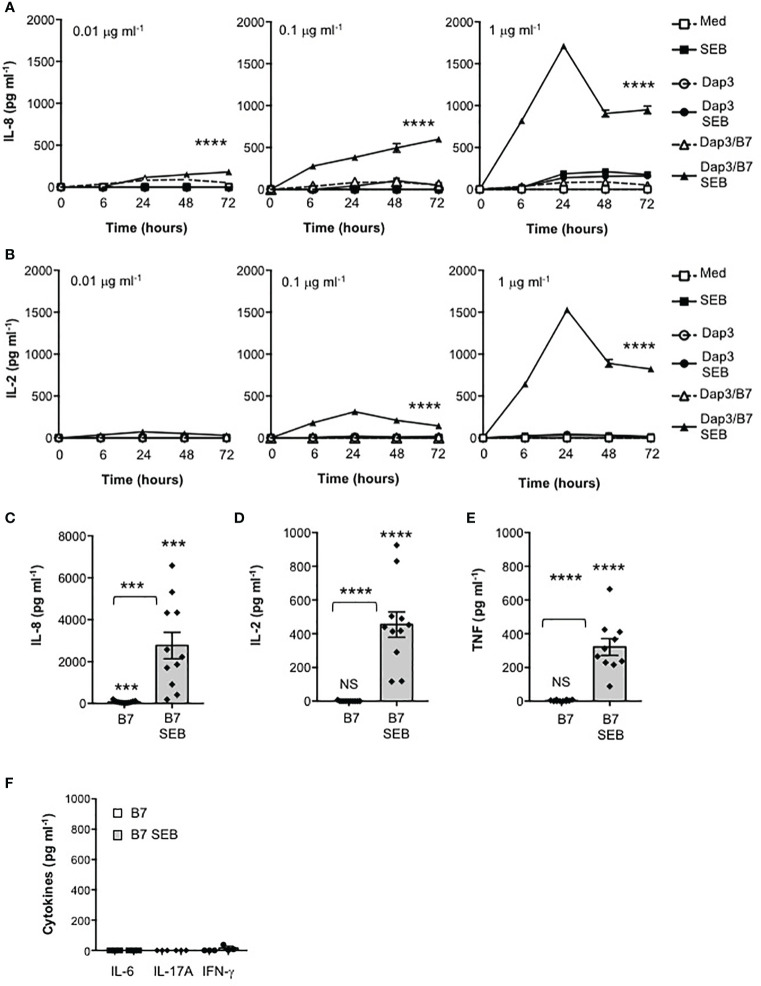
Stimulation of CD28 by SEB induces the secretion of pro-inflammatory cytokines and chemokines in the absence of MHC class II molecules. **(A, B)** CH7C17 Jurkat cells expressing human CD28WT were stimulated for the indicated times with murine L cells (Dap3) or Dap3 expressing human B7.1/CD80 (Dap3/B7) in the presence or absence of different doses of SEB. IL-8 **(A)** and IL-2 **(B)** levels in culture supernatant were measured by ELISA. Data show the mean ± SEM of one out of three independent experiments. Statistical significance was calculated by 2-way ANOVA test. **(C–F)** CD28WT cells were stimulated for 24 hours with Dap3/B7 in the absence or presence of 1 μg ml^-1^ SEB. **(C)** IL-8 (n = 11), **(D)** IL-2 (n = 11), **(E)** TNF (n = 10), **(F)** IL-6, IL-17A and IFN-γ (n = 3) levels in culture supernatant were measured by ELISA. Data show the mean ± SEM and statistical significance was calculated by Student’s *t* test. Means values: IL-8, medium (med) = 1.7 pg ml^-1^, B7 = 76 pg ml^-1^, B7 SEB = 2767 pg ml^-1^; IL-2, med = 0 pg ml^-1^, B7 = 0.6 pg ml^-1^, B7 SEB = 454.4 pg ml^-1^; TNF, med = 4.1 pg ml^-1^; B7 = 3.3 pg ml^-1^, B7 SEB = 321.6 pg ml^-1^; IL-6, med = 0 pg ml^-1^, B7 = 0 pg ml^-1^, B7 SEB = 0 pg ml^-1^; IL-17A, med = 0 pg ml^-1^, B7 = 0 pg ml^-1^, B7 SEB = 0 pg ml^-1^; IFN-γ, med = 0 pg ml^-1^, B7 = 0 pg ml^-1^, B7 SEB = 16.5 pg ml^-1^. (***) p < 0.001, (****) p < 0.0001, NS, not significant.

The excessive activation of cellular signalling pathways leading to inflammatory cytokine production by SEB relies on its ability to bind directly not only to specific TCR Vβ chains and MHC class II molecules (7, 9, 23) but also to CD28 and its ligands B7.1/CD80 or B7.2/CD86 (13–16). SEB is able to interact directly with CD28 and B7 molecules also in the absence of MHC class II or other coligands (13–16, 24), but the functional relevance of this interaction remains still unknown. Here, we examined the contribution of the CD28/B7 costimulatory axis on SEB-inflammatory cytokine production independently of MHC class II. To this end, we used a CD28-negative CH7C17 Jurkat T cell line expressing TCR Vβ3.1 (28) that specifically interacts with SEB (42) reconstituted with human CD28WT (29), and murine L cells (Dap3) or Dap3 cells expressing human B7.1/CD80 (Dap3/B7) (32). CD28WT and Dap3 cells did not express neither HLA-DR nor B7 molecules (**Supplementary Figures 1A, B**), whereas Dap3/B7 expressed only human B7.1/CD80 (**Supplementary Figure 1B**). The kinetic analysis of IL-8 (**Figure 1A**) and IL-2 (**Figure 1B**) secretion from CD28WT cells stimulated with different doses of SEB alone or in the presence of Dap3 or Dap3/B7 cells showed a strong and significant increase of both cytokines at all SEB doses only in the presence of Dap3/B7 cells (**Figures 1A, B**). Both cytokines reached maximal secretion after 24 hours of stimulation with Dap3/B7 and 1 μg ml^-1^ SEB (**Figures 1C, D**). Consistent with our previous data (21), the stimulation of CD28WT cells with B7.1/CD80-expressing cells induced a significant (p < 0.001 by Student’s *t* test) up-regulation of IL-8 secretion (mean = 76.1 pg ml^-1^) that was strongly enhanced by SEB (mean = 2767 pg ml^-1^) (**Figure 1C**). Analysis of the secretion of other pro-inflammatory cytokines produced by CD28WT cells stimulated for 24 hours with 1 μg ml^-1^ SEB in the presence of Dap3/B7 cells revealed a significant production of TNF (**Figure 1E**), whereas no secretion of IL-6, IL-17A and IFNγ was detected in culture supernatant (**Figure 1F**).

**Figure 2:**
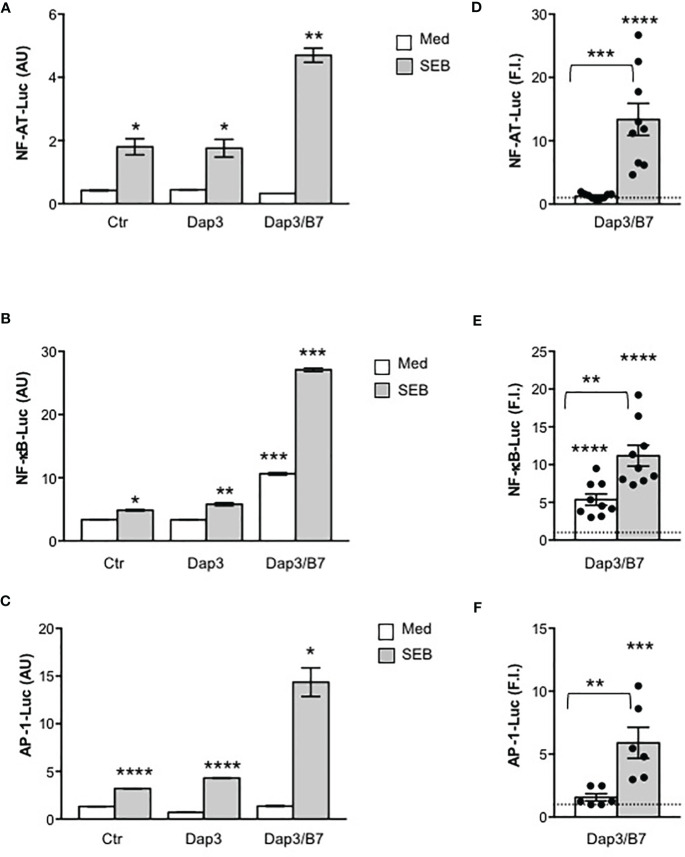
Stimulation of CD28 by SEB induces transcription factor activation in the absence of MHC class II molecules. **(A–F)** CD28WT cells transfected with 5 µg GFP together with 10 µg NF-AT-luciferase **(A, D)**, or 2 µg NF-κB-luciferase **(B, E)** or 10 µg AP-1-luciferase constructs **(C, F)** were unstimulated (Ctr) or stimulated for 6 hours with SEB (1 μg ml^-1^) alone or in the presence of Dap3 or Dap3/B7 cells. The results **(A–C)** are expressed as the mean of the luciferase units (AU) ± SEM after normalization to GFP expression and are representative of three independent experiments. **(D)** NF-AT luciferase activity (n = 9), **(E)** NF-κB luciferase activity (n = 9) and **(F)** AP-1-luciferase activity (n=8) of CD28WT cells stimulated with Dap3/B7 in the presence or absence of SEB. Bars show the mean fold induction (F.I) ± SEM after normalization to GFP values. Statistical significance was calculated by Student’s *t* test. Mean values: NF-AT, Dap3/B7 = 1.2, Dap3/B7 SEB = 13.3; NF-κB, Dap3/B7 = 5.3, Dap3/B7 SEB = 11.1; AP-1, Dap3/B7 = 1.5, Dap3/B7 SEB = 5.9. (*) p < 0.05, (**) p < 0.01, (***) p < 0.001, (****) p < 0.0001.

**Figure 3:**
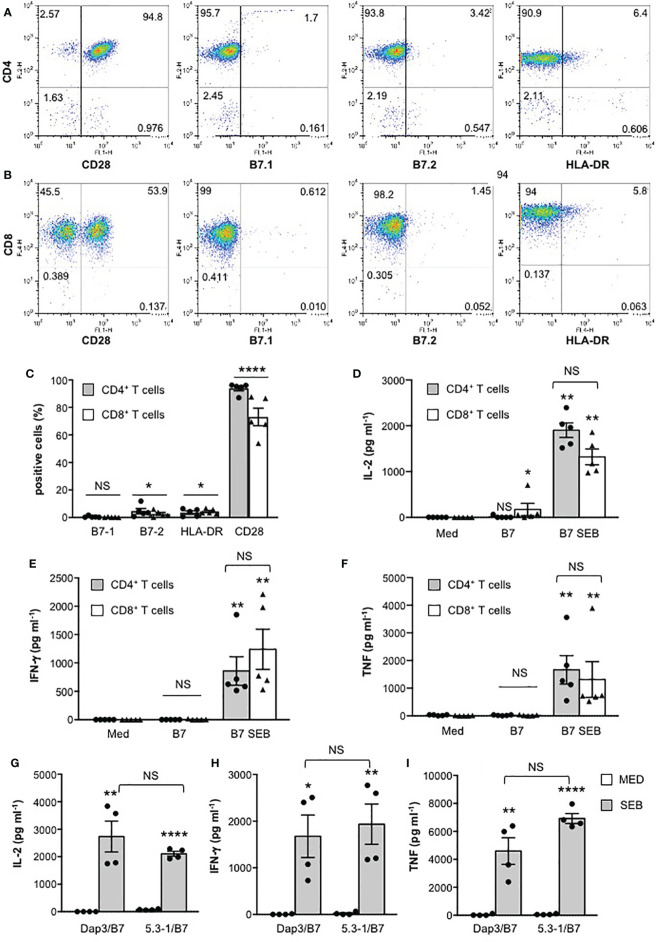
MHC class II molecules are dispensable for SEB-induced inflammatory cytokine production in human primary CD4^+^ and CD8^+^ T cells. **(A, B)** Human CD4^+^ T cells **(A)** or CD8^+^ T cells **(B)** were isolated from the peripheral blood of healthy donors (HD). One representative FACS analysis of human CD4^+^ T cells stained with anti-CD4-PE plus anti-CD28-FITC or anti-B7.1-FITC or anti-B7.2-FITC or anti-CD4-FITC plus anti-HLA-DR-APC **(A)**, or human CD8^+^ T cells stained with anti-CD8-APC plus anti-CD28-FITC, or anti-B7.1-FITC, or anti-B7.2-FITC, or anti-CD8-FITC plus anti-HLA-DR-APC **(B)**. Numbers within each quadrant indicate the percentage of positive cells. **(C)** The percentage of human CD4^+^ T cells or CD8^+^ T cells from HD (n = 5) expressing B7.1, B7.2, HLA-DR and CD28 were calculated. The results express the mean percentage of positive cells ± SEM. Statistical significance was calculated by Student’s *t* test. Mean percentage values: CD4^+^, B7.1 = 0.5, B7.2 = 4.6, HLA-DR = 3.5, CD28 = 93.8; CD8^+^, B7.1 = 0.2, B7.2 = 2.5, HLA-DR = 4.3, CD28 = 73. **(D–I)** CD4^+^ **(D–I)** or CD8^+^ T cells **(D–F)** from HD were cultured for 24 hours with medium (Med), or in the presence of Dap3/B7 cells **(D–I)** or 5-3.1/B7 cells **(G–I)** with or without 1 μg ml^-1^ SEB. IL-2 **(D)**, IFN-γ **(E)** and TNF levels **(F)** in culture supernatants were measured by ELISA. Data show the mean ± SEM. Statistical significance was calculated by the Mann-Whitney test. Mean values: **(D–F)** (n = 5) IL-2 CD4^+^, Med = 0.5 pg ml^-1^, B7 = 12.7 pg ml^-1^, B7 SEB = 1902 pg ml^-1^; IL-2 CD8^+^, Med = 0 pg ml^-1^, B7 = 173.4 pg ml^-1^, B7 SEB = 1320 pg ml^-1^; IFN-γ CD4^+^, Med = 0 pg ml^-1^, B7 = 0 pg ml^-1^, B7 SEB = 858.4 pg ml^-1^; IFN-γ CD8^+^, Med = 0 pg ml^-1^, B7 = 4.9 pg ml^-1^, B7 SEB = 1240 pg ml^-1^; TNF CD4^+^, Med = 24.8 pg ml^-1^, B7 = 19.4 pg ml^-1^, B7 SEB = 1664 pg ml^-1^; TNF CD8^+^, Med = 5.2 pg ml^-1^, B7 = 19.4 pg ml^-1^, B7 SEB = 1315 pg ml^-1^; **(G–I)** CD4^+^ T cells (n = 4), IL-2, Med = 0 pg ml^-1^, Dap3/B7 = 0 pg ml^-1^, Dap3/B7 SEB = 6036 pg ml^-1^, 5-3.1/B7 = 4.04 pg ml^-1^, 5-3.1/B7 SEB = 1006; IFN-g, Med = 1.1 pg ml^-1^, Dap3/B7 = 3.7 pg ml^-1^, Dap3/B7 SEB = 1676 pg ml^-1^, 5-3.1/B7 = 21.2 pg ml^-1^, 5-3.1/B7 SEB = 1935; TNF, Med = 8.9 pg ml^-1^, Dap3/B7 = 32.8 pg ml^-1^, Dap3/B7 SEB = 4592 pg ml^-1^, 5-3.1/B7 = 66.5 pg ml^-1^, 5-3.1/B7 SEB = 6920. (*) p < 0.05, (**) p < 0.01, (****) p < 0.0001. NS, not significant.

We next analysed the activation of the three major transcription factors regulating both IL-2 and other pro-inflammatory cytokine expression, NF-AT, AP-1 and NF-κB. In CD28WT cells, data from a representative experiment show that SEB alone was able to induce a significant activation of NF-AT (**Figure 2A**), NF-κB (**Figure 2B**) as well as AP-1 (**Figure 2C**). The presence of B7-negative Dap3 cells did not affect SEB-mediated upregulation of the three transcription factors (**Figures 2A–C**). By contrast, stimulation with Dap3/B7 cells strongly enhanced the activity of all three transcription factors induced by SEB (**Figures 2A–C**). Similar results were obtained using a larger sample size (**Figures 2D–F**). No significant differences in the activation of NF-AT (**Supplementary Figure 2B**), NF-κB (**Supplementary Figure 2C**) and AP-1 transcription factors (**Supplementary Figure 2D**) as well as on IL-2 (**Supplementary Figure 2E**) and IL-8 secretion (**Supplementary Figure 2F**) were observed when CD28WT cells were stimulated with SEB in the presence of 5.3-1/B7 cells that co-express human HLA-DRB1*0101 and B7.1/CD80 molecules (**Supplementary Figure 2A**), as compared to Dap3/B7 cells. Moreover, stimulation by SEB of primary CD4^+^ and CD8^+^ T cells, each expressing high levels of CD28 but very low levels of HLA-DR, B7.1 and B7.2 molecules (**Figures 3A–C**), in the presence of Dap3/B7 induced a strong and comparable production of IL-2 (**Figure 3D**), IFN-γ (**Figure 3E**) and TNF (**Figure 3F**). Similarly to CD28WT cells, no significant difference in IL-2 (**Figure 3G**), IFN-γ (**Figure 3H**) and TNF (**Figure 3I**) was observed when CD4^+^ T cells were stimulated with HLA-DR-positive 5-3.1 cells, as compared to Dap3/B7 cells. Hence, MHC class II molecules are dispensable for the activation of inflammatory signals by SEB, whereas the CD28/B7 costimulatory axis is pivotal.

### SEB Inflammatory Activity Requires Both TCR- and CD28-Mediated Signals

**Figure 4:**
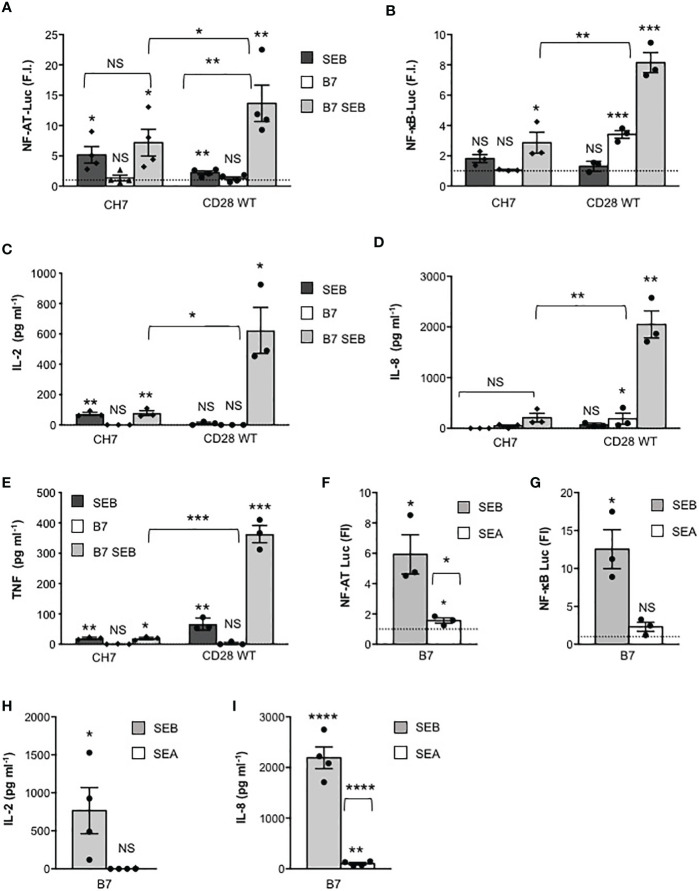
SEB-induced inflammatory activity requires CD28 signalling. **(A, B)** CD28-negative CH7C17 (CH7) or CD28WT Jurkat cells transfected with NF-AT luciferase **(A)** or NF-κB-luciferase constructs **(B)** were left unstimulated or stimulated for 6 hours with 1 μg ml^-1^ SEB in the presence or absence of Dap3/B7 cells (B7). Bars show the mean fold induction (F.I) ± SEM after normalization to GFP values. Statistical significance was calculated by Student’s *t* test. Mean values: NF-AT, CH7 SEB = 5.1, CH7 B7 = 1.3, CH7 B7 SEB = 7.1, CD28WT SEB = 2.2, CD28WT B7 = 1.2, CD28WT B7 SEB = 13.6; NF-κB, CH7 SEB = 1.8, CH7 B7 = 1, CH7 B7 SEB = 2.8, CD28WT SEB = 1.3, CD28WT B7 = 3.4, CD28WT B7 SEB = 8.1. **(C–E)** CH7 or CD28WT cells were cultured for 24 hours with medium (Med) or SEB in presence or absence of Dap3/B7 (B7) and IL-2 **(C)**, IL-8 **(D)** and TNF levels **(E)** in culture supernatants were measured by ELISA. Bars show the mean ± SEM of three independent experiments. Statistical significance was calculated by Student’s *t* test. Mean values: IL-2 CH7, Med = 0 pg ml^-1^, SEB = 72.8 pg ml^-1^, B7 = 0 pg ml^-1^, B7 SEB = 77.7 pg ml^-1^; IL-2 CD28WT, Med = 0 pg ml^-1^, SEB = 11.2 pg ml^-1^, B7 = 0 pg ml^-1^, B7 SEB = 622.9 pg ml^-1^; IL-8 CH7, Med = 0 pg ml^-1^, SEB = 46 pg ml^-1^, B7 = 0 pg ml^-1^, B7 SEB = 209.7 pg ml^-1^; IL-8 CD28WT, Med = 5.1 pg ml^-1^, SEB = 189.7 pg ml^-1^, B7 = 67.7 pg ml^-1^, B7 SEB = 2051 pg ml^-1^; TNF CH7, Med = 1.4 pg ml^-1^, SEB = 19.3 pg ml^-1^, B7 = 0.5 pg ml^-1^, B7 SEB = 19.1 pg ml^-1^; TNF CD28WT, Med = 1.1 pg ml^-1^, SEB = 66.3 pg ml^-1^, B7 = 3 pg ml^-1^, B7 SEB = 362.9 pg ml^-1^. NF-AT **(F)** and NF-κB **(G)** luciferase activities of CD28WT stimulated for 6 hours with 1 μg ml^-1^ SEB or SEA in the presence of Dap3/B7 cells (B7). The results were expressed as fold inductions (F.I.) over the basal level of luciferase activity in unstimulated cells. Data show the mean F.I. ± SEM after normalization to GFP expression. Statistical significance was calculated by Student’s *t* test. Mean values: NF-AT, B7 SEB = 5.928, B7 SEA = 1.559; NF-κB, B7 SEB = 12.54, B7 SEA = 2.313. **(H, I)** CD28WT cells were stimulated for 24 hours with SEB or SEA in presence of Dap3/B7 (B7). IL-2 **(H)** and IL-8 **(I)** levels in culture supernatants were measured by ELISA. Data show the mean ± SEM of four independent experiments. Significance was calculated by Student’s *t* test. Mean values: IL-2, Med = 0 pg ml^-1^, B7 SEB = 765.1 pg ml^-1^, B7 SEA = 0 pg ml^-1^ IL-8, Med = 0 pg ml^-1^, B7 SEB = 2191 pg ml^-1^, B7 SEA = 100.8 pg ml^-1^. (*) p < 0.05, (**) p < 0.01, (***) p < 0.001, (****) p < 0.0001, NS, not significant.

We next investigated the relative contribution of TCR and of CD28 to SEB-mediated activation of the signalling pathways regulating cytokine production. To this end, we analysed the response to SEB of CD28WT cells that express both CD28 and TCR/CD3 (**Supplementary Figure 1C**), CH7C17 cells (CH7) that express comparable levels of TCR/CD3 but not CD28 (**Supplementary Figure 1C**) and 31.13 Jurkat cells (30) that express a comparable level of CD28 but not of TCR/CD3 (**Supplementary Figure 1D**). Engagement of TCR by SEB on CD28-negative CH7 cells was sufficient to induce both Ca^2+^ influx (**Supplementary Figure 3**) and a significant increase in NF-AT transcriptional activity that did not change markedly in the presence of B7-expressing cells (**Figure 4A**). Consistent with the role of CD28 in enhancing TCR-induced Ca^2+^ influx and NF-AT activation (43), CD28/B7 costimulatory axis formation significantly increased SEB-induced Ca^2+^ (**Supplementary Figure 3**) and NF-AT activation in CD28WT cells (**Figure 4A**), as compared to CH7 cells. On the contrary, optimal NF-κB transcriptional activation by SEB was achieved only when both TCR and the CD28/B7 costimulatory axis were stimulated, as demonstrated by the strong increase observed in CD28WT cells stimulated by SEB in the presence of Dap3/B7 cells, as compared to CH7-stimulated cells (**Figure 4B**). Accordingly, SEB induced an optimal secretion of IL-2 (**Figure 4C**), IL-8 (**Figure 4D**) and TNF (**Figure 4E**) only when both the TCR and the CD28/B7 costimulatory axis were engaged. Indeed, SEA that binds CD28/B7 efficiently (24) but not TCRVβ3.1 (44) failed to activate either NF-AT (**Figure 4F**) or NF-κB (**Figure 4G**) and to stimulate the production of either IL-2 (**Figure 4H**) or IL-8 (**Figure 4I**).

**Figure 5:**
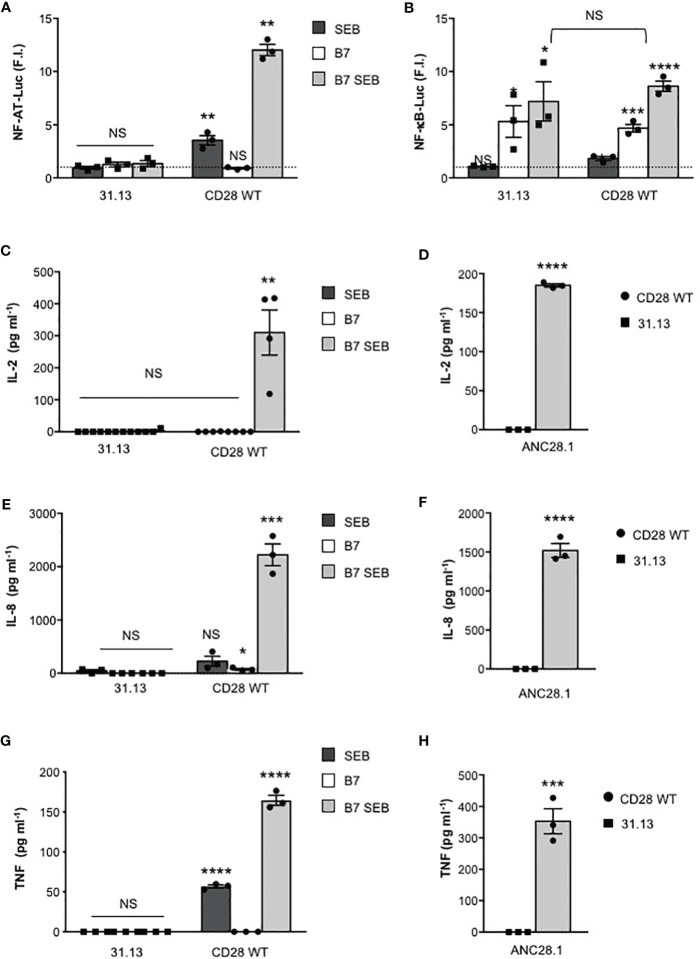
SEB inflammatory activity requires TCR signalling. **(A, B)** TCR-negative 31.13 and CD28WT cells were transfected with GFP together with NF-AT **(A)** or NF-κB luciferase constructs **(B)** and then stimulated with 1 μg ml^-1^ SEB in the presence or absence of Dap3/B7 cells (B7). Data show the mean fold induction (F.I.) ± SEM after normalization to GFP expression of three independent experiments. Statistical significance was calculated by Student’s *t* test. Mean values: NFAT, 31.31 SEB = 0.9, 31.31 B7 = 1.2, 31.13 B7 SEB = 1.3; CD28WT SEB = 3.5, CD28WT B7 = 0.9, CD28WT B7 SEB = 12; NF-κB, 31.13 SEB = 1, 31.13 B7 = 5.3, 31.13 B7 SEB = 7.2; CD28WT SEB = 1.8, CD28WT B7 = 4.6, CD28WT B7 SEB = 8.6. **(C–H)** 31.13 and CD28WT cells were unstimulated (Med) or stimulated for 24 hours with SEB in the presence or absence of Dap3/B7 cells (B7) or with 2 µg ml^-1^ ANC28.1 superagonistic anti-CD28 Abs. The levels of IL-2 **(C, D)**, IL-8 **(E, F)** and TNF **(G, H)** in culture supernatants were measured by ELISA. The result shows the mean ± SEM. Statistical significance was calculated by Student’s *t* test. Means values: 31.13, IL-2 Med = 0 pg ml^-1^, SEB = 0 pg ml^-1^, B7 = 0 pg ml^-1^, B7 SEB = 2,8 pg ml^-1^, ANC28.1 = 0 pg ml^-1^; CD28WT IL-2 Med = 0 pg ml^-1^, SEB = 0.4025 pg ml^-1^, B7 = 0 pg ml^-1^, B7 SEB = 310.2 pg ml^-1^, ANC28.1 = 185 pg ml^-1^; 31.31, IL-8 Med = 0 pg ml^-1^, SEB = 46 pg ml^-1^, B7 = 0 pg ml^-1^, B7 SEB = 0 pg ml^-1^, ANC28.1 = 0 pg ml^-1^; CD28WT, IL-8 Med = 1.35 pg ml^-1^, SEB = 225.9 pg ml^-1^, B7 = 73.9 pg ml^-1^, B7 SEB = 2221 pg ml^-1^, ANC28.1 = 1520 pg ml^-1^; 31.13, TNF Med = 0 pg ml^-1^, SEB = 0 pg ml^-1^, B7 = 0 pg ml^-1^, B7 SEB = 0 pg ml^-1^, ANC28.1 = 0 pg ml^-1^; CD28WT, TNF Med = 5.2 pg ml^-1^, SEB = 56.7 pg ml^-1^, B7 = 0 pg ml^-1^, B7 SEB = 164.5 pg ml^-1^, ANC28.1 = 353.3 pg ml^-1^. (*) p < 0.05, (**) p < 0.01, (***) p < 0.001, (****) p < 0.0001, NS, not significant.

The requirement of both TCR and CD28 for SEB-mediated T-cell activation was also confirmed in the TCR-negative 31.13 T cell line, where SEB stimulation was unable to induce any increase in NF-AT transcriptional activity (**Figure 5A**). Consistent with our previous data (45), in 31.13 cells, CD28 engagement by B7.1 strongly upregulated NF-κB transcriptional activity at levels comparable to those induced in CD28WT cells. However, in contrast to CD28WT cells, SEB did not induce any significant upregulation of NF-κB activity in 31.13 cells (**Figure 5B**). Similarly to CH7 cells, no IL-2 (**Figure 5C**), IL-8 (**Figure 5E**) or TNF secretion (**Figure 5G**) was induced by SEB stimulation of 31.13 cells. Moreover, in contrast to CD28WT (21), 31.13 cells were unable to secrete inflammatory cytokines when stimulated with ANC28.1 anti-CD28 superagonistic antibody (**Figures 5D, F, H**), thus supporting a role of TCR/CD3 in regulating CD28 signalling, as observed for CD2 (30).

**Figure 6:**
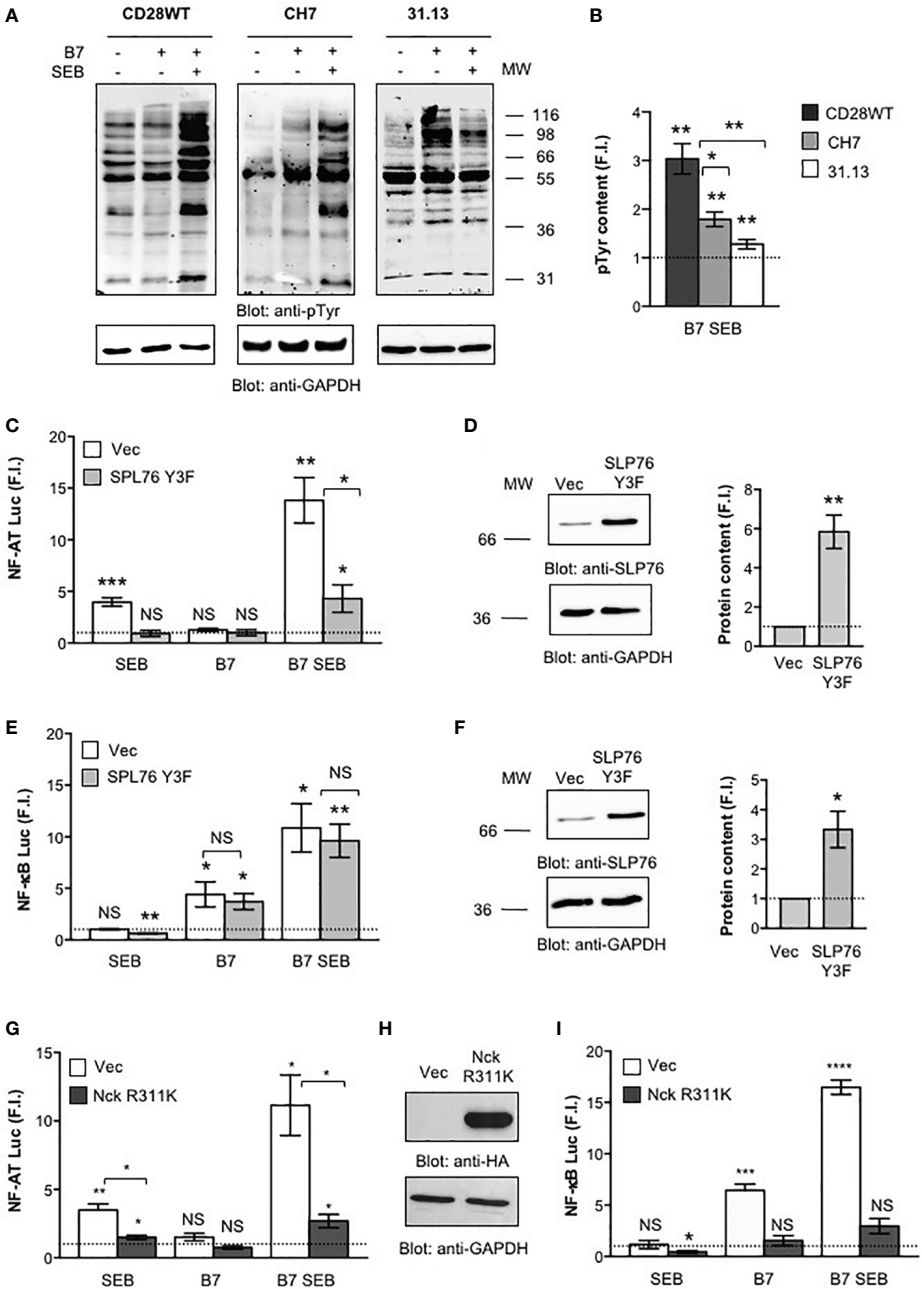
Phosphotyrosine and signalling mediators activated by both TCR and CD28 are required for SEB inflammatory activity. **(A, B)** CD28WT, CH7 and 31.13 cells were unstimulated or stimulated for 2 minutes with SEB in the presence or absence of Dap3/B7 cells (B7). Phosphotyrosine (pTyr) (upper panel) and GAPDH (lower) levels were analysed by western blotting **(A)** and quantified by densitometric analysis. pTyr content, normalized to GAPDH, was expressed as fold induction (F.I.) over the basal level of unstimulated cells **(B)**. The position of molecular weight (MW), expressed in kDa, is indicated on the right. Bars represent mean ± SEM of three independent experiments. Statistical significance was calculated by Student’s *t* test. **(C–I)** NF-AT **(C, G)** and NF-κB **(E, I)** luciferase activities of CD28WT cells transfected with empty vector (Vec) or Flag-SLP-76 Y3F mutant **(C–F)** or HA-Nck R311K **(G–I)** and activated for 6 hours with SEB in the presence or absence of Dap3/B7 (B7). The results were expressed as fold induction (F.I.) over the basal level of unstimulated cells transfected with Vec after normalization of GFP expression. Data show the mean ± SEM and statistical significance was calculated by Student’s *t* test. Anti-SLP-76 **(D, F)**, anti-HA **(H)** and anti- GAPDH western blotting was performed on total lysates. The position of molecular weight (MW), expressed in kDa, is indicated on the left. SLP-76 content **(D, E)**, normalized to GAPDH, was expressed as fold inductions (F.I.) over the basal level of unstimulated cells. Bars represent mean ± SEM and statistical significance was calculated by Student *t* test. Luciferase mean values: **(C, E)** NF-AT (n = 4), Vec SEB = 3.9, SLP-76 Y3F SEB = 0.9, Vec B7 = 1.2, SLP-76 Y3F B7 = 1, Vec B7 SEB = 13.8, SLP-76 Y3F = 4.3; NF-κB (n = 3), Vec SEB = 1.0, SLP-76 Y3F SEB = 0.6, Vec B7 = 4.4, SLP-76 Y3F B7 = 3.7, Vec B7 SEB = 10.8, SLP-76 Y3F = 9.6; **(G, I)** NF-AT (n = 3), Vec SEB = 3.4, Nck R311K SEB = 1.4, Vec B7 = 1.5, Nck R311K B7 = 0.7, Vec B7 SEB = 11.1, Nck R311K B7 SEB = 2.6; NF-κB (n = 3), Vec SEB = 31.1, Nck R311K SEB = 0.4, Vec B7 = 6.4, Nck R311K B7 = 1.5, Vec B7 SEB = 16.4, Nck R311K B7 SEB = 2.9. (*) p < 0.05, (**) p < 0.01, (***) p < 0.001, (****) p < 0.0001, NS, not significant.

We next analysed the early signalling events activated by both TCR and CD28 following SEB engagement and the contribution of key signalling mediators coupling TCR and/or CD28 to signalling pathways downstream. Tyrosine phosphorylation is the earliest signalling event activated following both TCR and CD28 co-engagement (43). Indeed, a strong increase in total phosphotyrosine content (pTyr) was induced by SEB in CD28WT cells stimulated with B7-expressing cells (**Figures 6A, B**). The absence of either TCR (31.13) or CD28 (CH7) significantly reduced pTyr levels following SEB stimulation (**Figures 6A, B**). The relative contribution of TCR and CD28 to SEB-induced activation of proximal signalling events was also analysed by evaluating the effects of SLP-76 and Nck signalling mediators. SLP-76 is a key substrate of TCR-activated tyrosine kinases (46, 47) that is required for the activation of several TCR-dependent signalling pathways (46, 48–50). Overexpression of a dominant-negative mutant of SLP-76 bearing mutations of Y113, Y128 and Y145 to phenylalanine (SLP-76 Y3F) (39) strongly impaired SEB-induced activation of NF-AT mediated by either TCR alone or TCR plus CD28/B7 costimulatory axis (**Figures 6C, D**). On the contrary, the activation of NF-κB that is mainly regulated by the CD28/B7 costimulatory axis (18) was not affected by overexpression of SLP-76 Y3F dominant-negative mutant (**Figures 6E, F**). Nck is an adapter molecule that plays a crucial role in the actin polymerization and cytoskeleton reorganization events required for T cell signalling and activation (51) and that can be recruited to either TCR (52–54) or CD28 (21). Overexpression of Nckβ R311K mutant within the SH2 domain (**Figure 6H**) that abrogates the interaction of Nck with CD28 (21) strongly impaired both NF-AT (**Figure 6G**) and NF-κB (**Figure 6I**) transcriptional activities induced by SEB. Altogether these results show that the TCR and CD28 cooperate in activating the signalling pathways regulating inflammatory cytokine expression in response to SEB stimulation in an MHC class II-independent manner.

### Binding of SEB to CD28 and B7.1 Favours T:APC Conjugate Formation and TCR Recruitment Into the Immunological Synapse

**Figure 7:**
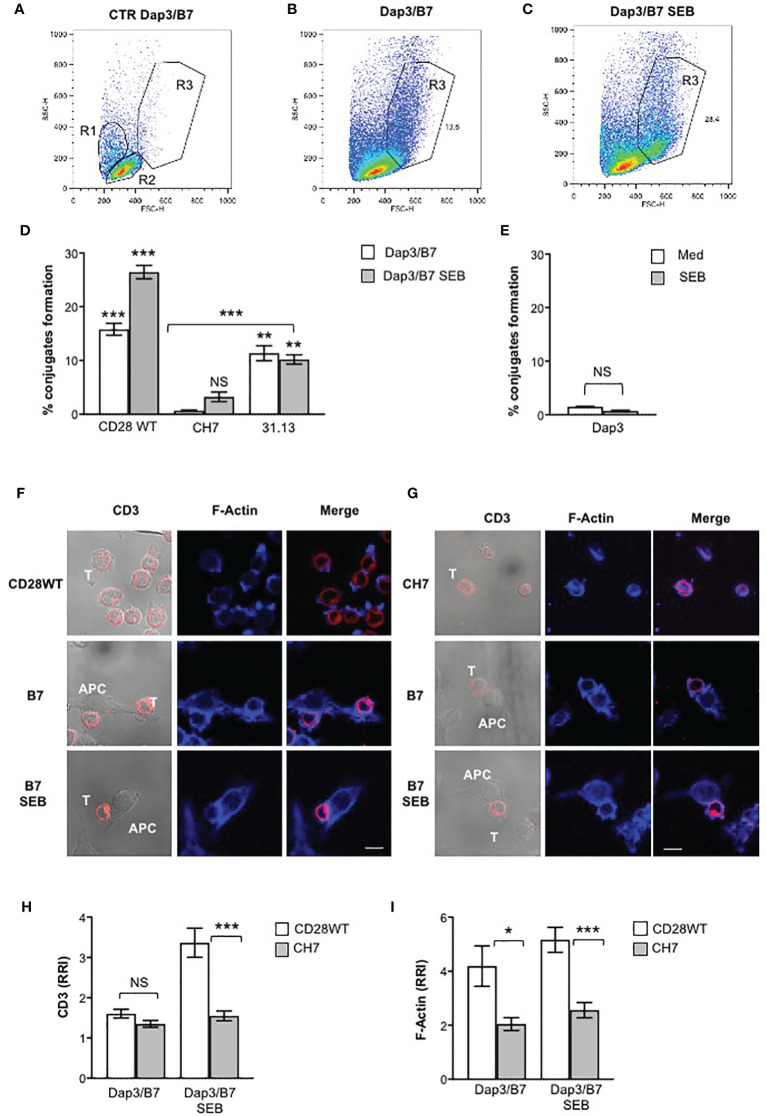
SEB increases T:APC conjugate formation and induces TCR recruitment into the immunological synapse. **(A, E)** CD28WT, CH7, and 31.13 cells were stimulated for 20 minutes with Dap3/B7 cells in the presence or absence of SEB and conjugate formation was analysed by FACS. **(A–C)** SSC vs FSC dot plots of CD28WT and Dap3/B7 cells either unconjugated **(A)** or conjugated for 20 minutes in the absence **(B)** or presence of SEB **(C)**. R1 and R2 gates **(A)** indicate the position of Dap3/B7 and CD28WT cells, respectively. Conjugates **(B, C)** were identified within the R3 gate and expressed as the mean percentage ± SEM of three independent experiments. **(D, E)** Conjugates formation between CD28WT, CH7, 31.13 cells **(D)** and Dap3 cells in the absence (Med) or presence of SEB expressed as the mean percentage ± SEM of three independent experiments. Statistical significance was calculated by Student’s *t* test. Mean percentage values: CD28WT, Dap3 = 1.5, Dap3 SEB = 0.8, Dap3/B7 = 15.8, Dap3/B7 SEB = 26,43; CH7, Dap3/B7 = 0.6, Dap3/B7 SEB = 3.2; 31.13, Dap3/B7 = 11.3, Dap3/B7 SEB= 10.1%. **(F–I)** CD28WT cells **(F)** or CH7 cells **(G)** were unstimulated or incubated with adherent Dap3/B7 (B7) in the presence or absence of SEB for 20 minutes. After fixing and permeabilization, CD3 (red) and F-actin (blue) were stained with anti-CD3 Ab (UCHT1) followed by Alexa 594-coniugated goat anti-mouse Ab and 633-conjugated phalloidin, respectively. The results **(F, G)** are representative of three independent experiments. The scale bar represents 10 µM. The relative recruitment index (RRI) of CD3 **(H)** and F-Actin **(I)** was calculated. Data represent the mean ± SEM of thirty-one conjugates analysed in each group. Statistical significance was calculated by Student’s *t* test. Mean values: CD3 CD28WT B7 = 1.6, CH7 B7 = 2.1, CD28WT B7 SEB = 3.4, CH7 B7 SEB = 1.7; F-Actin, CD28WT B7 = 4, CH7 B7 = 2, CD28WT B7 SEB = 5, CH7 B7 SEB = 2.5. (*) p< 0.05, (***) p < 0.001, (****) p < 0.0001. NS, not significant.

Most CD28 signalling functions rely on its capability to promote the actin reorganization events required for both T:APC interactions and TCR recruitment to the immunological synapse. Consistent with previous findings (19, 21, 22), both CD28WT and 31.13 cells formed conjugates (12-17%) when stimulated with Dap3/B7 cells, whereas CD28-negative CH7 cells did not (**Figures 7A–D**). Significantly, we found that SEB strongly increased the number of T:APC conjugates in CD28WT cells co-expressing both TCR and CD28 (24-27%), but not in 31.13 nor in CH7 cells (**Figure 7D**). By contrast, when B7-negative Dap3 cells were used, no significant conjugate formation (1.5%) was observed (**Figure 7E**), thus confirming the main role of CD28 in promoting cell-cell adhesion (19). These results indicate that, by binding simultaneously to TCR and CD28, SEB facilitates the recruitment of TCR to the T:APC contact zone, to induce an optimal signalling response. Accordingly, confocal analysis of CD3 recruitment and actin polymerization in CD28WT cells (**Figure 7F**) or CD28-negative CH7 cells (**Figure 7G**) conjugated with Dap3/B7 cells showed that SEB induced a strong recruitment of CD3 at the T:APC interface in CD28WT cells (**Figures 7F, H**) without affecting CD28-mediated F-actin accumulation (**Figure 7I**). On the contrary, in CH7 cells, SEB was not able to promote neither CD3 recruitment (**Figures 7G, H**) nor actin polymerization (**Figures 7G, I**).

**Figure 8:**
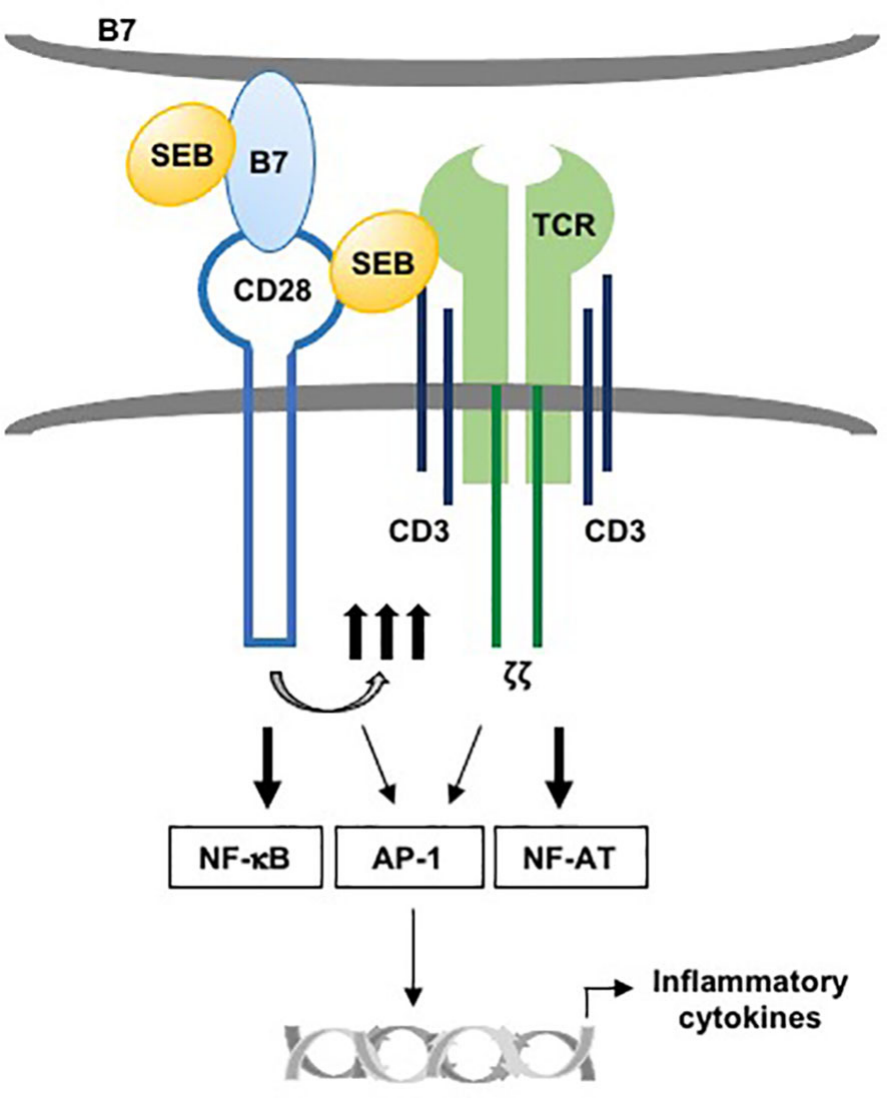
Schematic model of activation of TCR and CD28 by SEB in presence of B7. The binding of SEB to B7 as well as to TCR and CD28 promotes the activation of TCR and CD28 signalling. NF-AT is primarily regulated by TCR signalling, while NF-kB activation is under the control of CD28. AP-1 activation requires both TCR- and CD28-mediated signalling. The collaboration between TCR- and CD28/B7-mediated signalling in response to SEB leads to the production of pro-inflammatory cytokines.

Altogether, in line with the fact that SEB strongly promotes CD28/B7 engagement (15), these data indicate that binding of CD28 to B7, creating the costimulatory axis, is required for SEB-induced conjugate formation between T:APC and that TCR co-engagement by SEB results in more stable contacts that will favour optimal immunological synapse formation and hyperactivation of the inflammatory responses (**Figure 8**).

## Discussion

After the discovery of CD28 and its B7 coligands as critical receptors for SEB and other bacterial SAgs, the classical view of T-cell activation by SAgs has changed with a novel definition of the superantigen synapse, wherein the direct engagement of CD28 and B7 by SAgs in addition to TCR and MHC class II molecules is pivotal for the induction of inflammatory cytokines (13, 14, 16, 24–27). Here, we extend these data by showing that the MHC class II molecule is not required for SEB-mediated activation of T cells and by demonstrating a differential contribution of TCR and CD28.

For many years, it was thought that the binding of SEB to MHC class II is required to concentrate and stabilize SEB for recognition by the TCR (2). However, the formation of a binary complex between SEB and TCR may occur independently of MHC class II (8) and previous data by Goldbach-Mansky et al. showed that SEB can activate T cells in the absence of MHC class II if APCs expressed B7 molecules (55). More recent data by Levy et al. (16) and Popugailo et al. (24) demonstrated that SEB is able to directly interact with B7 molecules also in the absence of MHC class II molecules. These data together with the observation that the regions of SEB involved in binding B7 and TCR are distal (11, 16, 23, 24, 56), indicate that the binding of SEB to B7 molecules on APCs and to CD28 on T cells may suffice for a productive engagement of the TCR on T cells. Indeed, MHC class II-independent stimulation of T cells by SEB was previously reported, although the alternative SEB binding receptors were not identified (57–59). We demonstrated here that SEB efficiently activates inflammatory signalling in both TCR^+^CD28^+^ Jurkat T cells as well as in highly purified CD4^+^ and CD8^+^ T cells when presented by MHC class II-negative APCs expressing B7.1/CD80 (**Figures 1–3**), whereas the co-expression of MHC class II does not significantly enhance inflammatory signals mediated by TCR and CD28 neither in Jurkat cells (**Supplementary Figure 2**) nor in highly purified CD4^+^ T cells (**Figures 3G–I**). These data support a role for B7 as a pivotal receptor for SEB capable of initiating a productive binding interaction with both the TCR and CD28.

The TCR and CD28 cooperate to provide signals required for efficient T-cell activation (17, 18, 60). Following engagement by B7, CD28 acts as an amplifier of TCR signals in response to SEB by facilitating the initiation of TCR signals (17, 43, 61). On the other hand, CD28 signalling is also dependent on TCR signalling that upon stimulation causes an increase in local Ca^2+^ levels (62) making CD28 intracytoplasmic motifs accessible to signalling mediators (63), thus enhancing the calcium response (19, 29, 43, 64). Consistent with this concept, we found that TCR engagement by SEB was able to induce both Ca^2+^ (**Supplementary Figure 3**) and NF-AT activation (**Figure 4A**) also in the absence of CD28 engagement, whereas CD28 stimulation by SEB alone did not induce any change in either Ca^2+^ levels (**Supplementary Figure 3**) or NF-AT activity (**Figure 5A**). Although TCR-mediated Ca^2+^ increase and NF-AT activation were transient when compared to those induced when CD28 was co-engaged by SEB, these events depended exclusively on TCR signalling as demonstrated by the strong inhibition of NF-AT activation induced by overexpressing SLP-76 Y3F dominant-negative mutant (**Figure 6C**) (39). For instance, despite SLP-76 being a scaffold protein that regulates several TCR-activated signalling pathways (46, 48–50), it acts as a main regulator of the NF-AT signalling pathway by favouring the activation of PLCγ1 (65) as well as the nuclear import of NF-AT and its transcriptional activity (66, 67). By contrast, the SLP-76 Y3F dominant-negative mutant did not affect SEB-induced activation of NF-κB (**Figure 6E**) that is mainly regulated by CD28 (18) and depends on the recruitment of distinct adaptor signalling complexes (20, 21, 31, 45, 68–71).

Human CD28 is able to deliver TCR-independent signals by recruiting important signalling mediators, which leads to the activation of NF-κB (21, 22, 41, 71–73) and the expression of its target genes including pro-inflammatory cytokine and chemokine genes (20, 69, 70, 74, 75). Accordingly, CD28 engagement by B7.1 strongly upregulated NF-κB transcriptional activity also in the absence of the TCR (**Figure 5B**). These data, together with the inability of the TCR to activate NF-κB when engaged by SEB alone (**Figure 4B**), confirm the unique and pivotal role of CD28 in the activation of NF-κB (18, 76, 77). The ability of human CD28 to activate NF-κB in a TCR-independent manner relies on its C-terminal tyrosine phosphorylated YAPP motif that, once phosphorylated upon CD28 engagement by B7, binds the SH2 domain of Nck (21), an important regulator of the cytoskeleton reorganization events required for optimal T-cell activation (52, 53, 78). Indeed, overexpression of Nck mutant R311K within the SH2 domain strongly impaired NF-κB transcriptional activities induced by CD28/B7 interaction alone as well as by SEB-mediated TCR and CD28/B7 co-engagement (**Figure 6I**).

Another important issue that we addressed is whether TCR engagement by SEB may affect CD28/B7 interactions. Previous studies by Michel et al. clearly revealed a role of the CD28/B7 interaction in favouring the formation of the T:APC contact zone, thus facilitating TCR engagement by SEB and signalling (19). More recent data showed that SEB inflammatory activity relies on its ability to enhance the interaction of CD28 with both B7.1 and B7.2 (16, 24). Our data on conjugate formation in TCR-negative 31.13 cells clearly demonstrate that CD28/B7.1 interaction occurred also in the absence of the TCR but did not significantly changed when SEB was added (**Figure 7D**). On the contrary, the number of conjugates increased significantly (two-fold) when TCR and CD28 were co-engaged by SEB (**Figures 7C, D**), thus suggesting that SEB-induced TCR stimulation could enhance the CD28/B7 interaction. In this context, recent data from Sanchez-Lockart et al. highlighted that TCR stimulation by anti-CD3 antibodies activates an inside-out signalling that increases the avidity of CD28 for B7.1 by inducing the formation of a more stable CD28 homodimer interface, thus facilitating the bivalent binding to B7.1 (79–81), as well as optimal binding to SEB that specifically interacts with the same binding site within homodimer interface of CD28 (13). Thus, SEB may favour T:APC conjugate formation by acting as an intermolecular bridge between TCR and CD28, thus stimulating the TCR signals required for enhancing CD28/B7 interactions and formation of the immunological synapse. For instance, we found that CD3 was recruited to the T:APC contact zone and colocalized with polymerized actin following stimulation with B7.1-positive cells in CD28WT cells, but not in CH7 cells, and in the presence of SEB (**Figures 7F–I**). Experiments are in progress to analyse the dynamics of actin reorganization as well as TCR and CD28 recruitment at the IS in Jurkat cells interacting with lipid bilayers containing SEB and B7 molecules by using high-resolution total internal reflection fluorescence (TIRF) microscopy (82).

Altogether, our data revealed the dispensability of MHC class II molecules for SEB-mediated inflammatory responses, thus providing novel insights into the activation of T cells by SEB and other SAgs, which share the ability to bind CD28/B7 molecules, including diverse staphylococcal and streptococcal toxins (13, 16, 24) and the recently identified Spike protein of SARS-Cov2 (83). We propose a model wherein, by binding B7 molecules and simultaneously TCR and CD28, SAgs may recruit the TCR and CD28 into the immunological synapse, thereby triggering lethal inflammatory signalling (**Figure 8**).

## Data Availability Statement

The raw data supporting the conclusions of this article will be made available by the authors, without undue reservation.

## Ethics Statement

The studies involving human participants were reviewed and approved by Ethical Committee of the Policlinico Umberto I, Rome, Italy (ethical code N. 1061bis/2019, 13/09/2019). The patients/participants provided their written informed consent to participate in this study.

## Author Contributions

MK and CA performed most of the experiments, analysed the data, interpreted the results and helped in writing the manuscript. VT performed parts of the experiments and data analyses. SC contributed with technical support. MF contributed to discussions and helped in writing and editing the manuscript. RL, AP, and RK contributed to the design of experiments, discussions, writing and editing the manuscript. LT designed the study, coordinated the work, and wrote the manuscript. All authors contributed to the article and approved the submitted version.

## Funding

This work was supported by the Italian Foundation for Multiple Sclerosis (FISM 2016/R/29), “Progetto Ateneo” (Sapienza University of Rome, Italy) and Istituto Pasteur Italia-Fondazione Cenci Bolognetti (Sapienza University of Rome, Italy) to LT; “Progetto Ateneo” (Sapienza University of Rome, Italy) to MF; the Vigevani Fund (The Hebrew University of Jerusalem) to RK.

## Conflict of Interest

The authors declare that the research was conducted in the absence of any commercial or financial relationships that could be construed as a potential conflict of interest.

## Publisher’s Note

All claims expressed in this article are solely those of the authors and do not necessarily represent those of their affiliated organizations, or those of the publisher, the editors and the reviewers. Any product that may be evaluated in this article, or claim that may be made by its manufacturer, is not guaranteed or endorsed by the publisher.
