# Supplementary material for: Binding of Staphylococcal Enterotoxin B (SEB) to B7 Receptors Triggers TCR- and CD28-Mediated Inflammatory Signals in the Absence of MHC Class II Molecules
Source: Front Immunol. 2021 Aug 13;12:723689. doi: 10.3389/fimmu.2021.723689 (PMC8418141; doi:10.3389/fimmu.2021.723689)
Supplement: Supplementary file 1 [file Image_1.pdf]

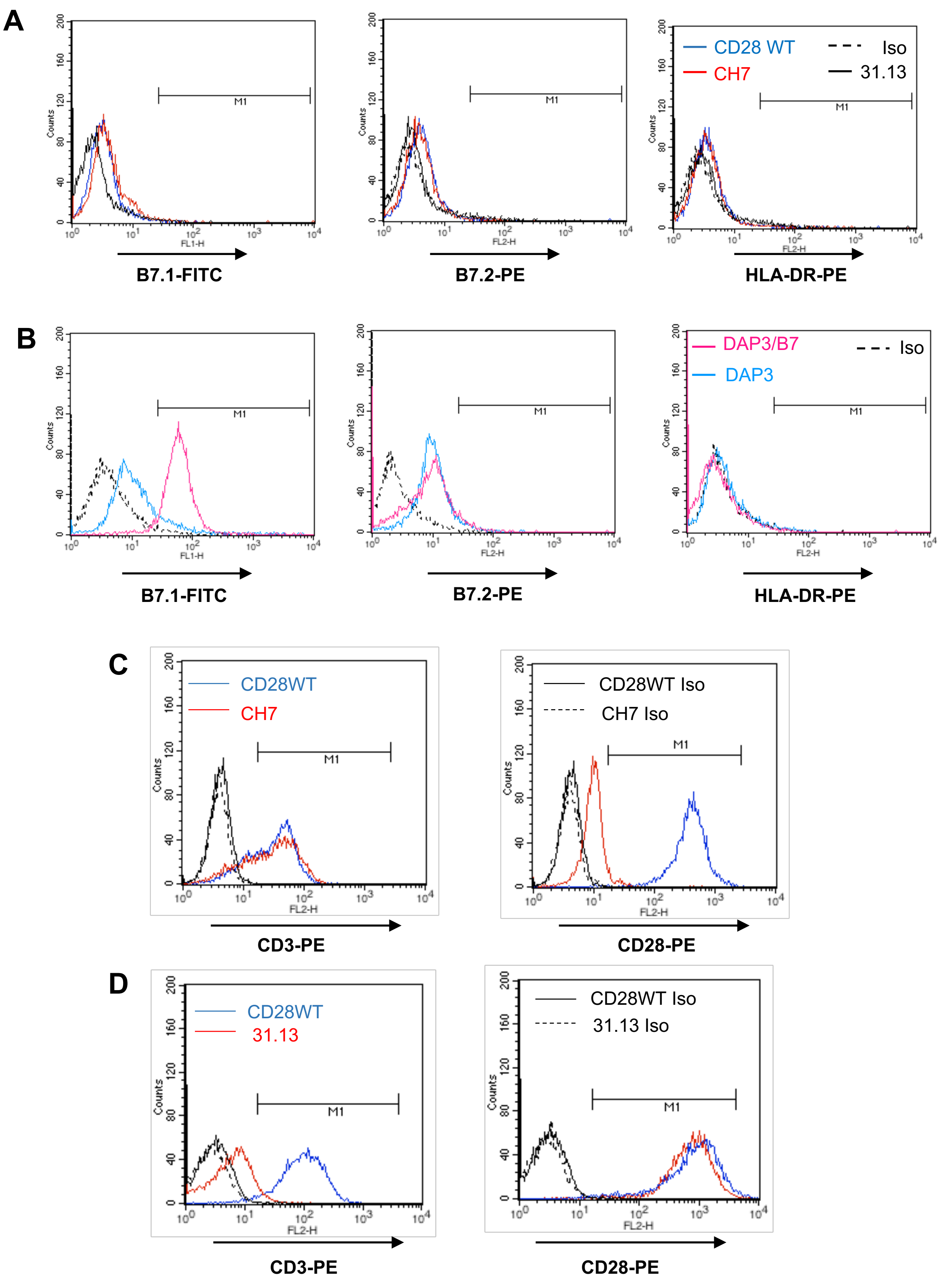

Figure S1. Phenotypic analysis of Jurkat T cell lines and murine L cells. (**A-D**) FACS analysis of B7.1, B7.2, HLA-DR (**A, B**), CD3 and CD28 (**C, D**) in CD28WT, CH7 and 31.13 cells or (**A, C, D**) or Dap3 and Dap3/B7 (**B**). Iso = isotype control Abs.
