# Supplementary material for: Binding of Staphylococcal Enterotoxin B (SEB) to B7 Receptors Triggers TCR- and CD28-Mediated Inflammatory Signals in the Absence of MHC Class II Molecules
Source: Front Immunol. 2021 Aug 13;12:723689. doi: 10.3389/fimmu.2021.723689 (PMC8418141; doi:10.3389/fimmu.2021.723689)
Supplement: Supplementary file 2 [file Image_2.pdf]

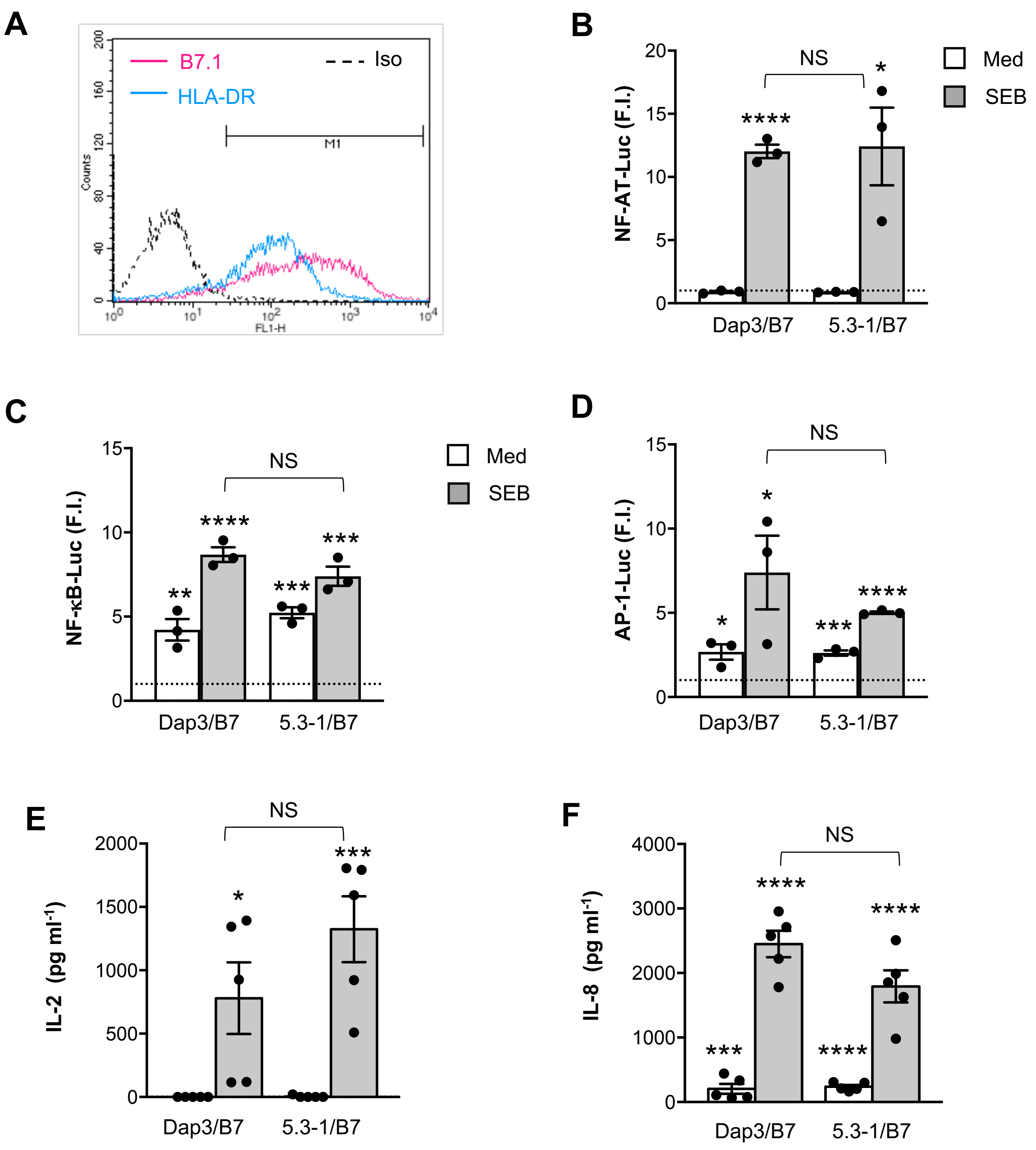

Figure S2. MHC class II contribution to SEB-mediated activation of NF-AT, NF-κB and AP-1 transcription factors. (**A**) FACS analysis of 5.3-1/B7 cells stained with FITC-conjugated isotype control (Iso), anti-B7.1-FITC Abs or anti-HLA-DR-FITC Abs. (**B-D**) CD28WT cells were transfected with 5 µg GFP together with 10 µg NF-AT (**B**) or 2 µg NF-κB (**C**) or 10 µg AP-1 (**D**) luciferase constructs and then stimulated for 6 hours with Dap3/B7 or 5.3-1/B7 cells in the absence (Med) or presence of 1 µg ml^{-1} SEB. Luciferase activity was measured and fold induction (F.I.) over the basal level of unstimulated cells was calculated after normalization of GFP values. Bars show the mean ± SEM and statistical significance was calculated by Student's *t* test. Mean values: NF-AT, Dap3/B7 = 0.9, Dap3/B7 SEB = 12, 5.3-1/B7 = 0.8, 5.3-1/B7 SEB = 12.4; NF-κB, Dap3/B7 = 4.2, Dap3/B7 SEB = 8.6, 5.3-1/B7 = 5.2, 5.3-1/B7 SEB = 7.3; AP-1, Dap3/B7 = 2.6, Dap/B7 SEB = 7.3, 5.3-1/B7 = 2.6, 5.3-1/B7 SEB = 5. (**E, F**) CD28WT cells were stimulated for 24 hours with Dap3/B7 or 5.3-1/B7 cells in the absence or presence of SEB. IL-2 (**E**) and IL-8 (**F**) levels in culture supernatant were measured by ELISA. Data show the mean ± SEM and statistical significance was calculated by Student's *t* test. Means values (pg ml^{-1}): IL-2, Dap3/B7 = 0, Dap3/B7 SEB = 420.7, 5.3-1/B7 = 4, 5.3-1/B7 SEB = 1000; IL-8, Dap3/B7 = 205.1, Dap3/B7 SEB = 2449, 5.3-1/B7 = 238.8, 5.3-1/B7 SEB = 1793. (*) $p < 0.05$, (**) $p < 0.01$, (***) $p < 0.001$, (****) $p < 0.0001$, NS = not significant.
