# Supplementary material for: Binding of Staphylococcal Enterotoxin B (SEB) to B7 Receptors Triggers TCR- and CD28-Mediated Inflammatory Signals in the Absence of MHC Class II Molecules
Source: Front Immunol. 2021 Aug 13;12:723689. doi: 10.3389/fimmu.2021.723689 (PMC8418141; doi:10.3389/fimmu.2021.723689)
Supplement: Supplementary file 3 [file Image_3.pdf]

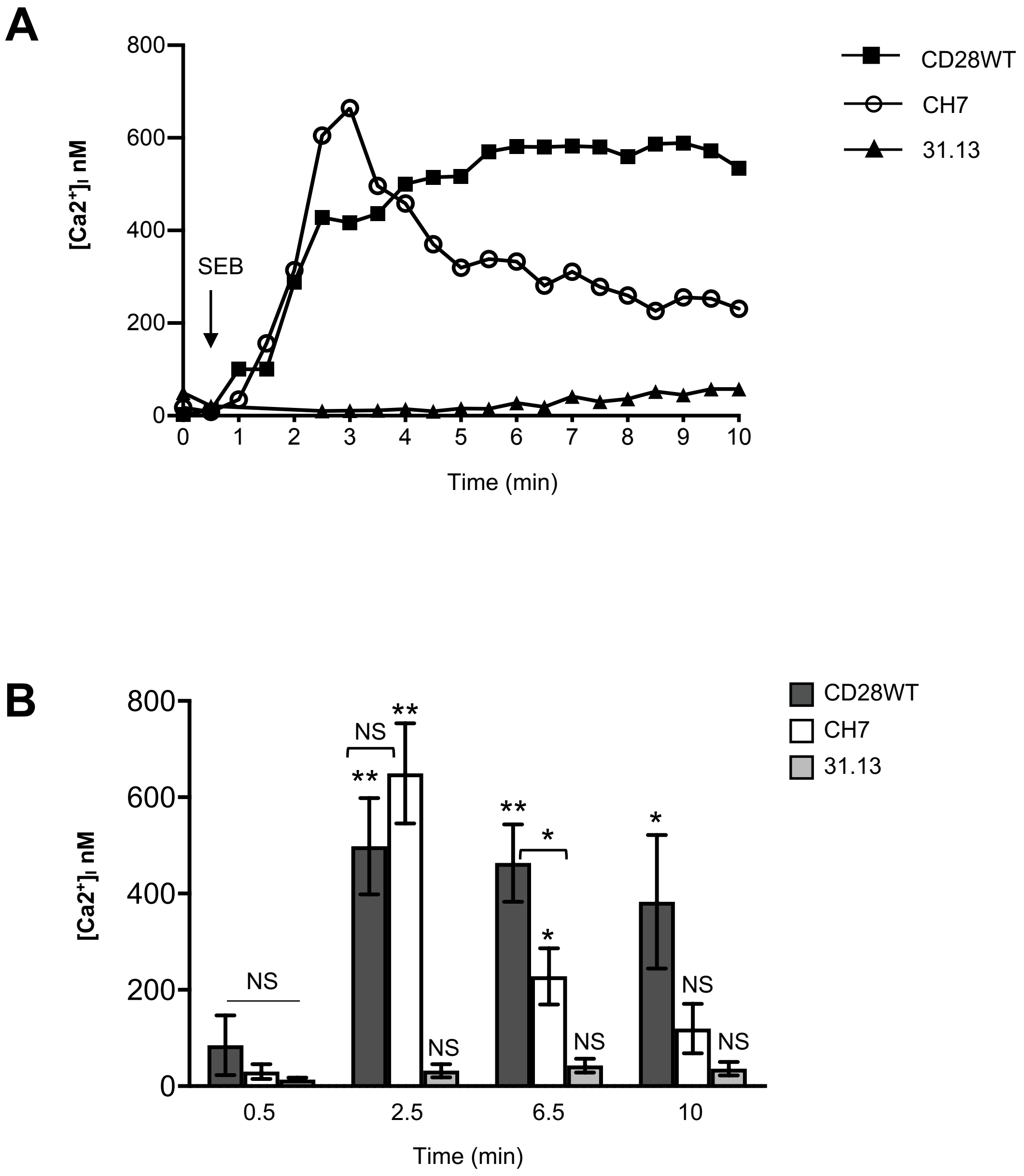

Figure S3. CD28 sustains SEB-induced calcium influx. (A, B) CD28WT, CH7 or 31.13 cells were loaded with Fluo-3-AM and then stimulated with 1 μg ml^{-1} SEB. Changes in Ca^{2+} levels ($[Ca^{2+}]_i$) were analysed by FACS every 30 s for 10 min by measuring the fluorescence emission in FL-1 channel. Data (**A**) are representative of three independent experiments. Bars (**B**) show the mean $[Ca^{2+}]_i$ ± SEM of three independent experiments at the indicated time points. Statistical significance was calculated by Student's t test.
